# The illusory perception of occluded space as empty depends on the occluded area

**DOI:** 10.1177/20416695251372334

**Published:** 2025-09-04

**Authors:** Pierre-Pascal Forster, Simon J. Hazenberg, Vebjørn Ekroll, Rob van Lier

**Affiliations:** 1 198328 Donders Institute for Brain, Cognition, and Behaviour, Radboud University, Nijmegen, The Netherlands; 2 87367 Department of Psychosocial Science, University of Bergen, Bergen, Norway This paper has been awarded the Early Career Best Paper Prize.

**Keywords:** illusion of absence, generic view principle, occlusion, obstruction of view, road accidents, magic, perception

## Abstract

Some occluders evoke the compelling impression that the space behind them is empty. Stage magicians use this illusion of absence to produce objects out of thin air. The generic view principle predicts that the illusion of absence should increase with decreasing occluder size. We investigated this prediction in experiments where participants saw a partly occluded scene and the same scene without the occluder, revealing a piece of fruit. They then rated (1) how easy it felt to imagine that the fruit was hidden behind the occluder and (2) how likely they thought it was that the fruit was hidden behind the occluder. Both ratings increased with increasing occluder area. This shows that the illusion of absence increases with decreasing occluder area, as predicted by the generic view principle. These findings could provide a starting point for future studies aiming to understand and prevent road accidents involving obstructions of view.

## How to cite this article

Pierre-Pascal Forster, Simon Jan Hazenberg, Vebjørn Ekroll, Rob Van Lier. (2025). The illusory perception of occluded space as empty depends on the occluded area. *i-Perception*, *16*(5), 1–12

## Introduction

**Figure 1.**
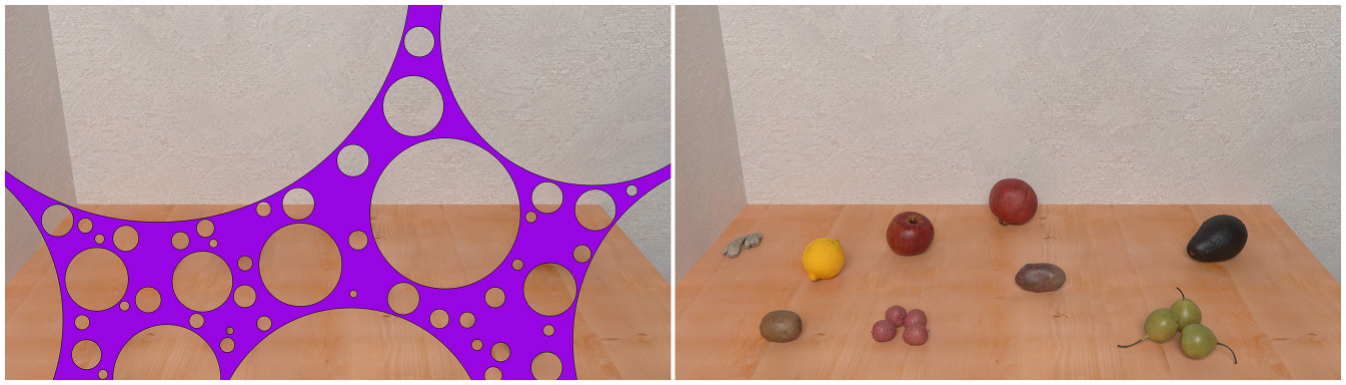
In this illustration of the illusion of absence, the space behind the purple occluder on the left looks empty. Nevertheless, the occluder can hide many different objects in the covered space, as can be seen in the right image. Knowing that these objects are hidden behind the occluder does not prevent the impression that the space behind the occluder looks empty.

Although objects in our environment frequently become invisible due to occlusion, we generally assume that they continue to exist (Flombaum et al., 2008; Teichmann et al., 2022). Even infants who are only a few months old are able to experience object permanence (Baillargeon et al., 1985). Due to our strong sense of object permanence, we are thoroughly surprised when magicians make things (seemingly) vanish into thin air or appear out of nowhere. It has been suggested that the illusion of absence (Figure 1) plays an important role in creating this type of magical experiences (Ekroll et al., 2017; Svalebjørg et al., 2020). In the illusion of absence, some occluding objects readily provoke the impression that the space behind them is empty (Ekroll et al., 2021). Thus, cleverly hiding an object in one's hand can lead to the impression that the object has magically disappeared (Ekroll et al., 2017; Kuhn & Land, 2006).

The illusion of absence, underlying many magic tricks, might also affect human behaviour in other settings. For example, the illusion of absence may contribute to road accidents if another road user is hidden in a blindzone they perceive as empty, such as behind one of the A-pillars supporting the car's roof on both sides of the windscreen (Ekroll et al., 2021). An analysis of video-recorded accidents in Hong Kong suggests that A-pillar occlusion is an important contributory factor to road accidents (Cheng et al., 2019). Scientific knowledge about the mechanisms underlying the illusion of absence and stimulus conditions triggering it may be crucial for understanding and preventing this type of accident.

The mechanisms underlying this illusion are so far unknown, but one well-known theoretical framework that could potentially explain the illusion of absence is the generic view principle (Albert, 2001). According to this principle, the visual system assumes that the basic layout (topological structure) of the retinal image would not change when the visual scene is viewed from a slightly different angle. In Figure 1, the total occlusion of another object requires a highly coincidental alignment along the line of sight, and even a small change in the viewpoint would reveal the objects from behind the occluder, which would lead to a qualitative change in the retinal image. Thus, based on the generic view principle, the visual system should assume the interpretation of an object with the given size hidden behind the occluder, to be highly unlikely. When keeping the size of the hidden object constant, larger occluders allow for more possibilities where changes in viewpoint preserve the invisibility of the hidden object (and hence the qualitative structure of the retinal image). Conversely, smaller occluders make it more difficult for an object of the given size to fit behind the occluder, leading to a stronger illusion of absence (Ekroll et al., 2021). The illusion of absence could thus depend on the occluded area, the relative size of the occluded area and the hidden object, or both. When seeing an occluded scene, the visual system has no direct access to what might be hidden behind the occluder, which could suggest that the occluded area is the most prominent factor driving the illusion of absence. In this study, we investigate if the illusion of absence depends on the occluded area.

Some support for the hypothesis that the illusion of absence depends on the occluded area comes from a study by Øhrn et al. (2019). Two limitations of their study that we address in the present investigations, however, are (1) that they only investigated two levels of the occluder size and (2) that they measured the illusion of absence indirectly using a levitation criterion, rather than more directly as in the present study.

In this study, we provide empirical evidence for the illusion of absence by investigating how the occluded area modulates participants’ perception of empty space. In two experiments presenting stimuli either simultaneously or sequentially, participants rated questionnaire statements probing their perception of occluded space. Based on the generic view principle (Albert, 2001), we hypothesised that the illusion of absence becomes stronger with decreasing occluder area (Ekroll et al., 2021; Øhrn et al., 2019). We further expected both questionnaire statements to correlate positively. To anticipate, our results clearly support the prediction based on the generic view principle.

## Methods

### Participants

We recruited 48 participants (41 females, 7 males) via the online platform Sona Systems (Sona Systems Ltd., Tallinn, Estonia). Participants had a mean age of 22 (*SD* = 8) years. As indicated by the Edinburg Handedness Inventory (EHI, Oldfield, 1971), most participants were right handed (EHI ≥60: 33 participants; EHI ≤ −60: 6 participants; 60 > EHI > −60: 9 participants). Two participants stated that they had an uncorrected refractive error, but did not need to use glasses regularly. Participants participated either in an experiment using a sequential presentation mode or in an otherwise identical experiment using a simultaneous presentation mode (24 participants each). They received 10€ or course credits as compensation. The study is in line with the Declaration of Helsinki 2013 and was approved by the local ethics committee. All participants gave written informed consent prior to the experiment.

### Stimuli and Task

**Figure 2.**
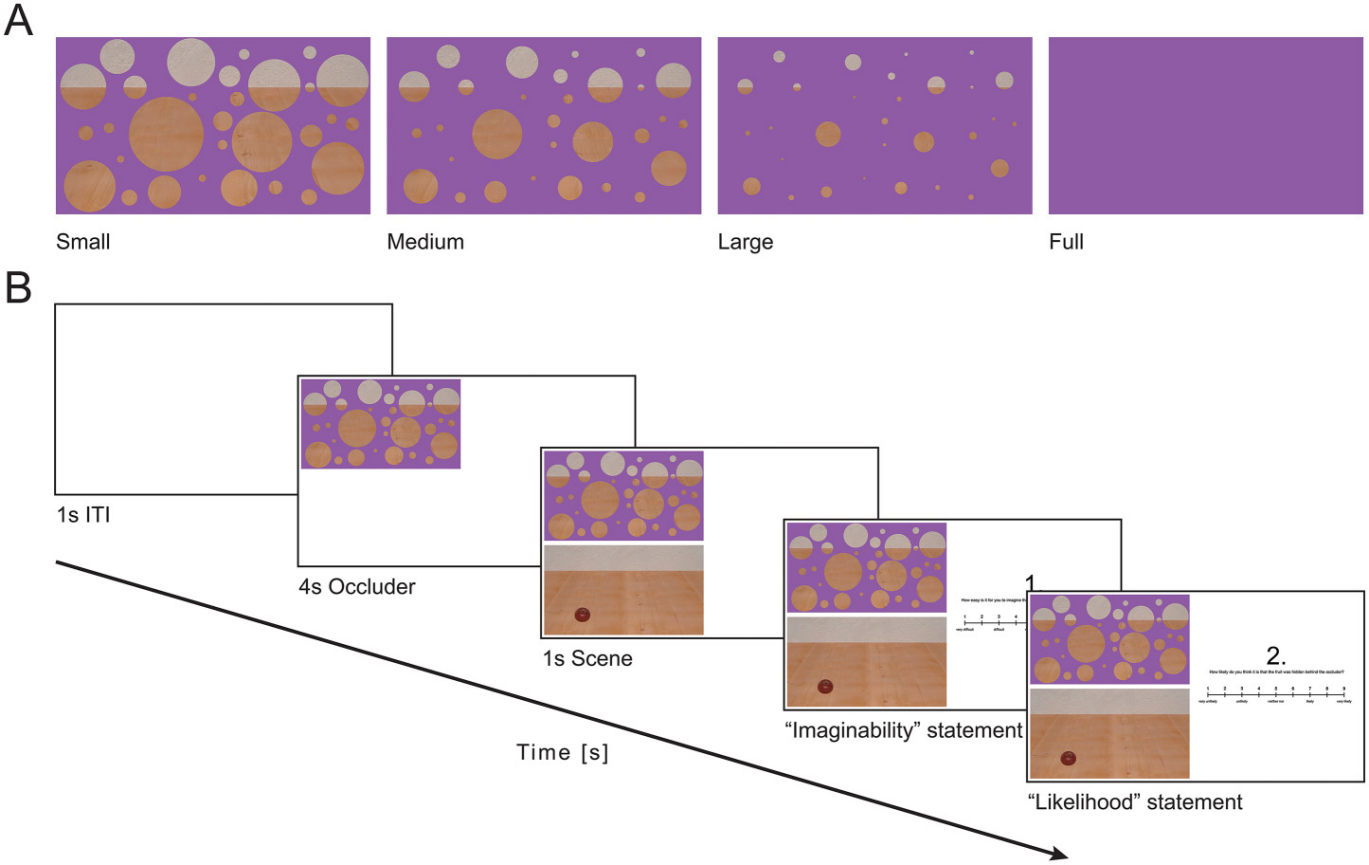
(A) The area of the occluder in the stimuli increased from small to full. (B) In the experiment using the simultaneous presentation mode, participants first saw a blank screen (ITI) and then an occluder stimulus at the top left of the screen. Subsequently, the scene without the occluder appeared at the bottom left. At the end of each trial, participants rated two questionnaire statements while both stimuli remained on screen. During the sequential presentation mode (not shown in the figure), occluder and scene stimuli both appeared at the centre left side of the screen with the scene stimulus covering the occluder stimulus after it appeared.

We created the occluder stimuli (Figure 2A) in Adobe Illustrator 2024 (Adobe Inc., San Jose, CA, USA) based on a previous illustration of the illusion of absence (Ekroll et al., 2017, p. 98). We manipulated the occluder area by decreasing the size of the holes in the occluder through which parts of the background scene were visible. Small, medium, large and full occluders covered 54.31%, 79.46%, 94.69% and 100% of the background scene, respectively. For each of these levels of occluder area, except the latter, we created three different configurations of occluder stimuli that differed in the position of the holes. The background scene was created in Blender (Stichting Blender Foundation, Amsterdam, The Netherlands) and contained a piece of fruit (apple, pear or lemon) rendered without shadows from a single viewpoint and placed in one of six positions in Adobe Illustrator.

Immediately after the presentation of each stimulus pair, participants responded to the following two questionnaire statements: “*How easy is it for you to imagine that the fruit was hidden behind the occluder?*” (henceforth “imaginability statement”) and “*How likely do you think it is that the fruit was hidden behind the occluder?*” (henceforth “likelihood statement”). To do so, they pressed a button on the keyboard corresponding to the value on a nine-point Likert scale ranging from one (very difficult/very unlikely) to nine (very easy/very likely).

### Design

We ran two experiments with slightly differing trial structures (between-subject factors). In both experiments, participants first saw an occluder stimulus. The succeeding scene stimulus appeared below the occluder stimulus in the simultaneous presentation mode (Figure 2B), and at the same position in the sequential presentation mode. Thus, both occluder and scene stimuli were visible when rating the questionnaire statements in the simultaneous presentation mode but only the scene stimulus when doing the ratings in the sequential presentation mode. The two experiments were otherwise identical.

We balanced the order of the questionnaire statements across participants and randomised the order of the presentation of the trials within participants. Each experiment consisted of 180 trials per participant, with a 1-minute break every 60 trials. In a repeated measures-design, participants saw the full occluder on 18 trials (6 positions × 3 fruits) and the small, medium and large occluders on 54 trials each (3 fruits × 6 positions × 3 configurations). Both experiments were conducted using PsychoPy 2023.2.3 (e.g., Peirce, 2009) using Python 3.8.

### Procedure

We established the participants’ dominant hand using the Edinburg Handedness Inventory (EHI, Oldfield, 1971) and asked them to use this hand to respond. If participants indicated to use both hands or a mixture of both hands in several statements, we asked them to use their preferred hand instead.

Before starting the experiment, participants familiarised themselves with the task by performing two practise trials. Each trial started with an intertrial interval (ITI) of 1 s, consisting of a white screen (Figure 2B). Participants then saw one of the occluder stimuli for 4 s, followed by an image of the same scene without the occluder, showing a piece of fruit that had been hidden behind the occluder for 1 s. Depending on the experiment, this scene stimulus appeared below (simultaneous presentation mode) or at the same position as the occluder stimulus (sequential presentation mode). After seeing both occluder and scene stimuli, participants rated two questionnaire statements (see Stimuli and Task) by pressing a button on the keyboard.

### Preprocessing and Analysis

Preprocessing was done in Python 3.12. Very short response times likely reflect accidental button presses rather than valid responses. To take this into account, we marked ratings with a log response time of more than 3 times the standard deviation below the mean as outliers (0.74% of the “imaginability” ratings and 0.5% of the “likelihood” ratings) and excluded the respective values before fitting the model. Additionally, for two participants we recorded two key presses at the same time for one of the “imaginability” ratings and excluded these responses from the analysis.

We analysed the data in R 4.4.3 by fitting a Bayesian ordinal multilevel model with a probit link to the data using the brms package (v2.22.0, Bürkner, 2017). This model assumes that participants’ ordinal responses stem from a normally distributed latent variable that was segmented into discrete response categories (Bürkner & Vuorre, 2019; Liddell & Kruschke, 2018). We combined the data from both experiments in one model. For each questionnaire statement we fitted a model with occluder area, presentation mode and their interaction as fixed effects. To account for the dependence in the data, we added a random intercept for each participant. We used a treatment coding with the small occluder and the sequential presentation mode as reference categories. Rather than assuming a linear relationship between the occluder area and participants’ ratings, this model allows for arbitrary differences between any two occluder area levels. We further calculated the correlation between the “imaginability” and “likelihood” ratings as the residual correlation from a multivariate random intercept model using a student's *t*-distribution, without additional predictors. All models used brms’ default priors and were run with 10,000 iterations and four chains. All reported 95% credible intervals are based on percentiles. Tables with all model results can be found in the Supplemental Material A.

In an additional analysis, we calculated the probability that a piece of fruit could be hidden behind the different occluders. We calculated this for the whole occluder image and did not restrict our calculation to areas of the table. In this way, we can estimate how probable it is that a randomly placed object is hidden anywhere behind the occluder. In 100 draws for each stimulus combination, we randomly placed a piece of fruit onto the occluder and evaluated the percentage of cases where it was completely occluded. We simulated 100 of these samples for each stimulus combination and report the mean and the standard deviation of the percentages of fully occluded fruits.

## Results

**Figure 3.**
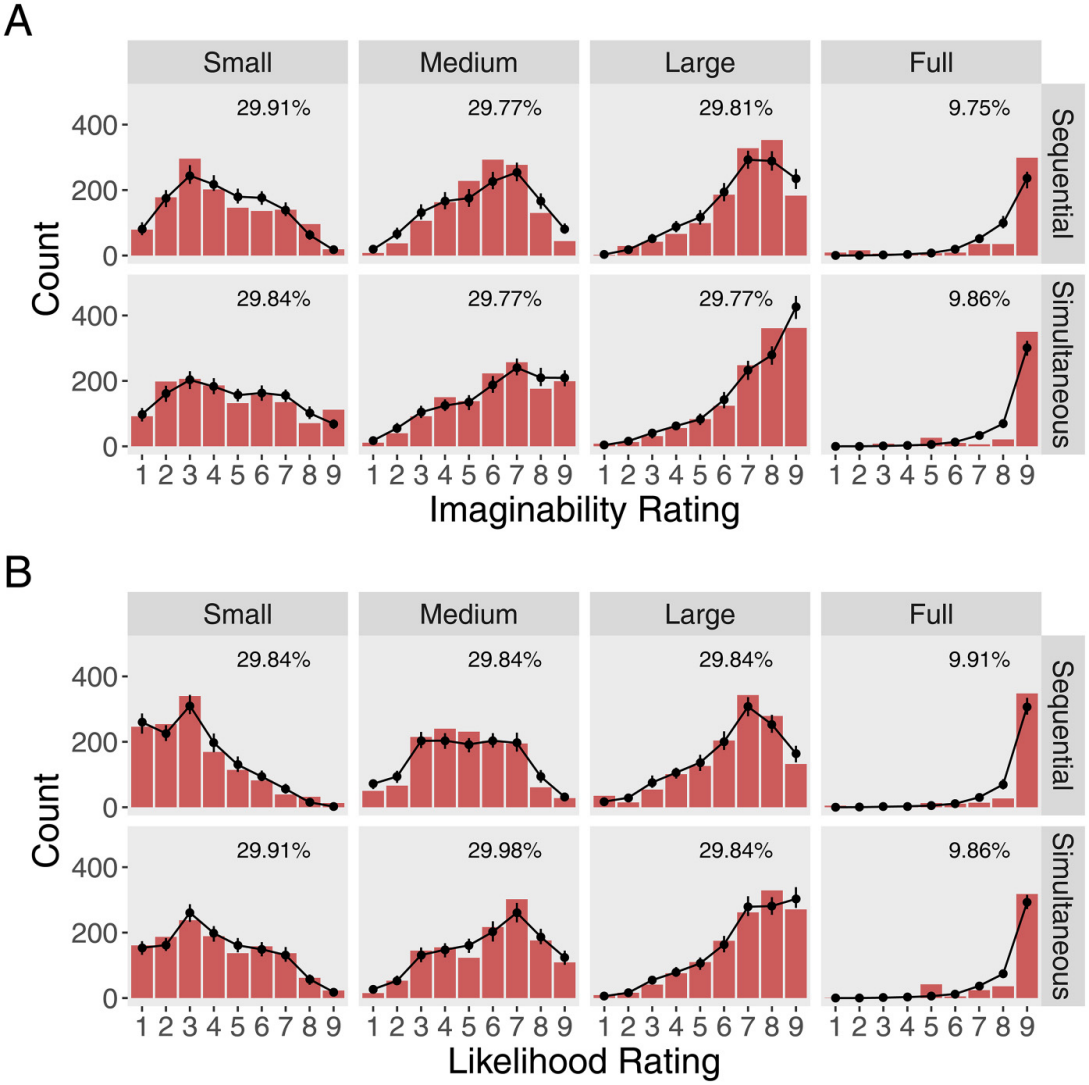
To illustrate the model fit, (A) shows count data and model draws from the predictive distribution for the “imaginability” and (B) for the “likelihood” ratings. Red bars show the count data of participants' responses for each occluder area and presentation mode. The black dots show the mean count and the 95% credible interval for 100 draws from the posterior predictive distribution. Percentages in the subplots indicate the number of valid trials per presentation mode.

Figure 3A shows the distributions of “imaginability” ratings for each combination of occluder condition (small, medium, large or full) and presentation mode (sequential or simultaneous). Figure 3B shows the same for the “likelihood” ratings. Comparing draws from the posterior predictive distribution (black dots) to the counts of participants’ responses (red bars) shows that our models fit the empirical data well.

**Figure 4.**
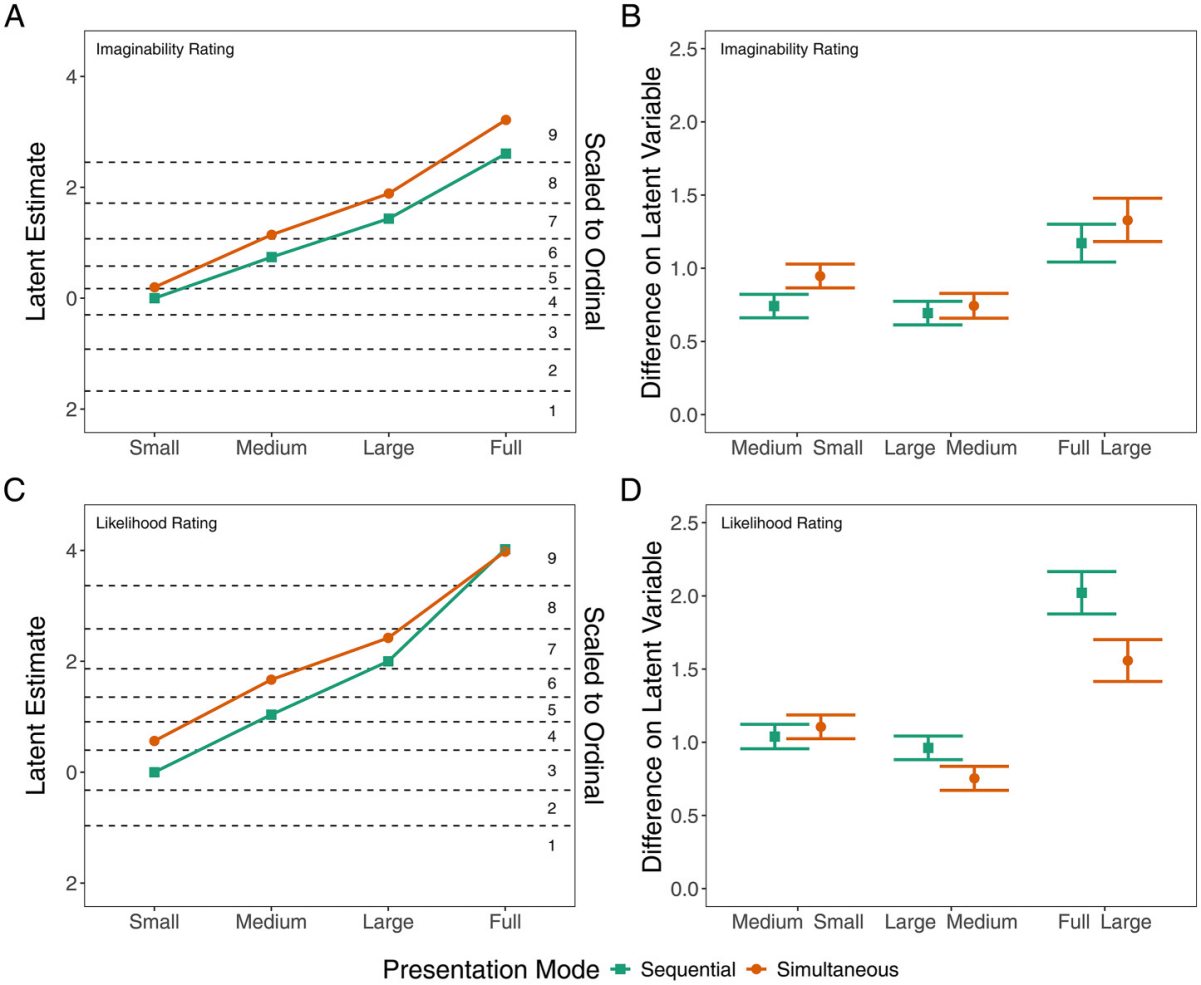
(A) The model estimates for the “imaginability” rating on a latent scale for each occluder area and presentation mode. Dashed lines represent the model's thresholds between the response categories 1–9, which are indicated on the right side of the figure. (B) The differences in the “imaginability” rating in terms of the model's latent variable between successive occluder areas for both presentation modes. Positive values indicate higher ratings in the larger of the two compared occluder area levels. Error bars represent the 95% credible interval. (C) and (D) show the same as (A) and (B), but for the “likelihood” rating rather than the “imaginability” rating.

Figure 4A and C show the models’ posterior means on a latent scale and how these values translate into ordinal response categories for the “imaginability” and “likelihood” ratings, respectively. Comparing each consecutive level of occluder area for each presentation mode showed that participants’ ratings increased monotonically with increasing occluder area in both the sequential and the simultaneous presentation modes, for both questionnaire statements. This means that participants found it easier to imagine, and more likely, that something was hidden behind larger occluders than smaller ones, independent of the presentation mode. Figure 4B and D plot the posterior mean differences between the occluder areas on the latent scale with corresponding 95% credible intervals, and Table 1 lists the corresponding numerical values.

**Table 1.** Comparison between successive occluder areas.

	Medium–small		Large–medium		Full–large	
Statement	Sequential	Simultaneous	Sequential	Simultaneous	Sequential	Simultaneous
Imaginability	0.74 [0.66, 0.82]	0.95 [0.87, 1.03]	0.69 [0.61, 0.77]	0.74 [0.66, 0.83]	1.17 [1.04, 1.30]	1.33 [1.18, 1.48]
Likelihood	1.04 [0.96, 1.12]	1.11 [1.02, 1.19]	0.96 [0.88, 1.04]	0.75 [0.67, 0.84]	2.02 [1.88, 2.17]	1.56 [1.42, 1.70]

*Note.* Estimates of the difference between ratings in successive occluder area levels on the latent scale for each presentation mode. Values in brackets show the 95% credible interval. Positive values indicate higher ratings in the larger of the two compared occluders.

We further compared the effect of presentation mode over the different occluder areas and observed minor differences between presentation modes. As can be seen in Figure 4A and C and Figure S1 in Supplemental Material B, participants tended to give somewhat higher “imaginability” and “likelihood” ratings in the simultaneous presentation mode. However, the 95% credible intervals of the estimates of the difference (see Table 2 and Supplemental Figure S1) excluded zero only for the large and full occluders for the “imaginability” statement and only for the small and medium occluders for the “likelihood” statement.

**Table 2.** Comparison between presentation modes.

Statement	Small	Medium	Large	Full
Imaginability	0.20 [-0.25, 0.62]	0.40 [-0.04, 0.84]	0.45 [0.01, 0.89]	0.61 [0.13, 1.07]
Likelihood	0.56 [0.09, 1.04]	0.63 [0.16, 1.11]	0.42 [-0.05, 0.90]	-0.04 [-0.54, 0.46]

*Note.* Estimates of the difference between ratings in the different presentation modes per occluder area. Values in brackets show the 95% credible interval. Positive values indicate higher ratings in the simultaneous compared to the sequential presentation mode.

Furthermore, as is evident from comparing Figure 4A and C, the model results are highly similar for the imaginability and the likelihood ratings. This is in line with a large positive correlation between the “imaginability” and “likelihood” ratings for both presentation modes (sequential: *r* = .78 [.77, .80]; simultaneous: *r* = .84 [.83, .85]; 95% credible interval in brackets), suggesting that both questionnaire statements measure similar constructs.

To quantify how probable it is that a randomly placed piece of fruit would be occluded by the occluders in our stimuli, we ran an additional analysis by randomly placing a piece of fruit onto the occluder stimulus and calculating the percentage of fully occluded fruit placements. On average, only 0.48% (*SD* = 0.70) of the random placements were fully occluded behind the small occluder, compared to 7.31% (*SD* = 2.84) for the medium, 30.73% (*SD* = 5.01) for the large, and 100% for the full occluder. Restricting the occluder area to where a fruit could plausibly occur gave highly similar results. These results mirror participants’ ratings and emphasise the accidentality of the viewpoint required for an object to be fully occluded behind smaller occluders.

## Discussion

In two experiments presenting stimuli either sequentially or simultaneously, we investigated if the illusion of absence depends on the occluded area. As predicted by the generic view principle (Albert, 2001) and in line with the findings by Øhrn et al. (2019), the results showed that participants’ “imaginability” and “likelihood” ratings increased with increasing occluder area, which suggests that participants perceive a stronger illusion of absence with smaller occluders, where larger hidden objects barely fit behind the occluder.

The highly similar results for both questionnaire statements, also reflected in their high correlation, suggest that both measure the same construct. However, an alternative interpretation could be that participants attempted to keep their ratings internally consistent, anchoring their second response on the first (Gehlbach & Barge, 2012). While this could contribute to the high correlation between questionnaire statements, it cannot explain why participants’ ratings increased with increasing occluder area, which is the main finding of this study.

An advantage of our study compared to the previous investigation by Øhrn et al. (2019) is the more direct measurement of the illusion of absence. As a downside, this may make the experimental purpose more transparent to participants and hence increase the risk of biases associated with demand characteristics (Orne, 1962). This makes it in principle possible that participants responded based on perceived experimental demands, possibly trying to respond in favour of the researcher's hypotheses (Corneille & Lush, 2023). However, the converging evidence with the study by Øhrn et al. (2019) suggests that both studies measure the same underlying construct and that the illusion of absence generalises across experimental designs. Nevertheless, to rule out potential weaknesses due to demand characteristics, the next step should be to extend our design to measure the illusion of absence more indirectly, for example using electroencephalography (EEG).

If the illusion of absence induces the perception that the occluded space is empty, no objects should be expected to be hidden in the occluded space. This expectation might be encoded in a predictive coding framework, where revealing an unexpected object from behind an occluder could lead to a prediction error, that is, a surprise response (e.g., Huang & Rao, 2011). The amount of surprise when a hidden object is revealed should then be positively related to the strength of the illusion of absence. This prediction could be captured by measuring participants’ P3 response in EEG that can be associated with surprise when revealing objects from behind an occluder (Hazenberg & van Lier, 2016). In line with this general idea, Baykan and Schütz (2025) reported that disoccluding objects leads to a P3 response, if the scene is relatively empty, but the prediction needs to be tested more directly based on behavioural measurements of the strength of the illusion of absence such as the ones provided by the present study. Thus, the results we report here provide an important basis for further research on the illusion of absence and its neural correlates.

In all trials in our experiment, a piece of fruit was revealed to be hidden behind the occluder, which may be expected to influence participants expectations over time, and hence also their “imaginability” and “likelihood” ratings. However, comparing participants ratings from the first half of the experiment against the second half revealed only minor differences (Supplemental Material C). In addition, we observe a clear effect of occluder area, even though participants likely learned that a piece of fruit would occur behind all occluders equally likely, likewise arguing against a purely cognitive response strategy. This is in line with previous findings (Svalebjørg et al., 2020) suggesting that the illusion of absence is cognitively impenetrable in much the same way as perceptual processes at large (Firestone & Scholl, 2016; Pylyshyn, 1999). Men et al.'s (2023) finding that the number of occluded objects are underestimated relative to straightforward expectations based on the number of visible unoccluded objects are also in line with the general idea that humans cannot help but to perceive the space behind certain types of occluders as empty despite conflicting conscious knowledge.

It is reasonable to assume that the illusion of absence falls apart as soon as parts of the hidden object become visible, leading to amodal completion of the partially occluded object (e.g., van Lier & Gerbino, 2015). Nevertheless, it seems that both the illusion of absence and amodal completion can be connected to the generic view principle (as interpretations of partly occluded shapes tend to minimize coincidental alignments, van Lier et al., 1994).

The results we present here are largely independent of the paradigm, showing that both presentation modes are equally well suited to further investigate the illusion of absence. The slight differences between presentation modes could reflect different strategy uses or memory loads. It is for example conceivable that participants based their ratings on a cognitive size matching strategy in which they compared the presented piece of fruit to the space behind the occluder, which might be more prominent in the simultaneous presentation mode. This makes it difficult to completely separate perceptual and cognitive processes in our experiment. Therefore, future research needs to distinguish these processes involved in participants’ judgements (c.f., Kanizsa, 1985).

Regardless of the strategy used, erroneously judging an object to not fit behind an occluder is of high practical relevance, for example in road traffic. In road traffic, drivers need to correctly anticipate where other road users may appear to avoid accidents. For example, perceiving the space behind the A-pillar as empty might not only hinder road users from checking the blindzone, but could also deter the anticipation of potential other road users on collision course and therefore slow down reactions to other road users suddenly emerging from the occluded space (Ekroll et al., 2021). It is therefore important for future research to investigate more directly whether the illusion of absence affects road safety, for example, by using driving simulations in virtual reality.

In summary, the results we present here show that the illusion of absence gets stronger as the occluder covers less of the background. Furthermore, these results generalise across different presentation modes. This clearly supports the hypothesis that the generic view principle underlies the illusion of absence. These insights into this newly described illusion are not only of high theoretical relevance, but presumably also of practical importance, for example, as a starting point for new experiments for understanding and preventing road accidents involving obstructions of view. Based on the present results, one may expect that relatively narrow obstructions of view, such as the A-pillars in cars, are more likely to evoke the potentially misleading impression that the blindzone behind it is empty than wider obstructions of view.

## Supplemental Material

sj-pdf-1-ipe-10.1177_20416695251372334 - Supplemental material for The illusory perception of occluded space as empty depends on the occluded area Supplemental material, sj-pdf-1-ipe-10.1177_20416695251372334 for The illusory perception of occluded space as empty depends on the occluded area by Pierre-Pascal Forster, Simon J. Hazenberg, Vebjørn Ekroll and Rob van Lier in i-Perception
