# Supplementary material for: The illusory perception of occluded space as empty depends on the occluded area
Source: Iperception. 2025 Sep 4;16(5):20416695251372334. doi: 10.1177/20416695251372334 (PMC12411715; doi:10.1177/20416695251372334)
Supplement: sj-pdf-1-ipe-10.1177_20416695251372334 - Supplemental material for The illusory perception of occluded space as empty depends on the occluded area [file sj-pdf-1-ipe-10.1177_20416695251372334.pdf]

Supplementary material

The illusory perception of occluded space as empty depends on the occluded area

A: Model results

Table S1

Model output "imaginability" rating

Component	Estimate	Error	CI_{lower}	CI_{upper}	R-hat	Bulk ESS	Tail ESS
SD Intercept	0.75	0.08	0.61	0.92	1.00	2,416.08	4,824.65
Intercept 1	-1.67	0.16	-1.99	-1.37	1.00	2,232.99	3,997.56
Intercept 2	-0.92	0.16	-1.23	-0.62	1.00	2,188.97	3,921.98
Intercept 3	-0.30	0.16	-0.61	0.00	1.00	2,175.59	4,090.16
Intercept 4	0.17	0.16	-0.13	0.47	1.00	2,182.90	4,034.08
Intercept 5	0.58	0.16	0.27	0.88	1.00	2,173.87	3,984.88
Intercept 6	1.07	0.16	0.77	1.37	1.00	2,188.43	3,922.14
Intercept 7	1.71	0.16	1.41	2.02	1.00	2,214.04	3,828.69
Intercept 8	2.45	0.16	2.14	2.76	1.00	2,229.53	4,093.00
$Occluder_{M-S}$	0.74	0.04	0.66	0.82	1.00	10,480.08	13,232.47
$Occluder_{L-S}$	1.43	0.04	1.35	1.52	1.00	10,104.95	13,119.57
$Occluder_{F-S}$	2.61	0.07	2.47	2.74	1.00	11,523.51	12,367.18
$Presentation_{Sim-Seq}$	0.20	0.22	-0.25	0.62	1.00	2,296.01	4,255.25
$Occluder_{M-S}$: $Presentation_{Sim-Seq}$	0.21	0.06	0.09	0.32	1.00	11,184.54	13,245.17
$Occluder_{L-S}$: $Presentation_{Sim-Seq}$	0.25	0.06	0.14	0.37	1.00	11,076.33	12,703.90
$Occluder_{F-S}$: $Presentation_{Sim-Seq}$	0.41	0.10	0.22	0.61	1.00	12,158.94	13,548.31

Note. Estimates and errors indicate the mean and standard deviation of the posterior distribution, CI_{lower} and CI_{upper} display the lower and upper limits of the 95% credible interval.

Table S2

Model output "likelihood" rating

Component	Estimate	Error	CI_{lower}	CI_{upper}	R-hat	Bulk ESS	Tail ESS
SD Intercept	0.80	0.08	0.66	0.99	1.00	2,617.01	3,999.68
Intercept 1	-0.97	0.17	-1.30	-0.63	1.00	2,254.59	4,549.63
Intercept 2	-0.32	0.17	-0.66	0.00	1.00	2,236.36	4,486.05
Intercept 3	0.40	0.17	0.07	0.73	1.00	2,240.01	4,483.30
Intercept 4	0.91	0.17	0.58	1.24	1.00	2,243.92	4,377.77
Intercept 5	1.35	0.17	1.02	1.68	1.00	2,245.54	4,407.40
Intercept 6	1.87	0.17	1.54	2.20	1.00	2,242.08	4,471.56
Intercept 7	2.59	0.17	2.25	2.92	1.00	2,270.64	4,447.70
Intercept 8	3.36	0.17	3.03	3.70	1.00	2,297.08	4,516.67
$Occluder_{M\text{-}S}$	1.04	0.04	0.96	1.12	1.00	10,482.34	12,974.67
$Occluder_{L\text{-}S}$	2.00	0.04	1.91	2.09	1.00	10,436.77	13,690.45
$Occluder_{F\text{-}S}$	4.02	0.08	3.87	4.18	1.00	10,963.72	12,573.79
$Presentation_{Sim\text{-}Seq}$	0.56	0.24	0.09	1.04	1.00	2,333.03	4,398.53
$Occluder_{M\text{-}S}$: $Presentation_{Sim\text{-}Seq}$	0.07	0.06	-0.05	0.18	1.00	11,273.99	12,721.61
$Occluder_{L\text{-}S}$: $Presentation_{Sim\text{-}Seq}$	-0.14	0.06	-0.26	-0.03	1.00	10,866.53	13,344.88
$Occluder_{F\text{-}S}$: $Presentation_{Sim\text{-}Seq}$	-0.61	0.10	-0.81	-0.40	1.00	11,464.20	12,626.77

Note. Estimates and errors indicate the mean and standard deviation of the posterior distribution, CI_{lower} and CI_{upper} display the lower and upper limits of the 95% credible interval.

Table S3

Output correlation model sequential presentation mode

Component	Estimate	Error	CI_{lower}	CI_{upper}	R-hat	Bulk ESS	Tail ESS
SD Intercept Likelihood	1.13	0.18	0.84	1.55	1.00	5,312.69	8,216.13
SD Intercept Imaginability	0.87	0.14	0.65	1.19	1.00	5,005.28	6,366.84
Intercept Likelihood	5.18	0.24	4.71	5.65	1.00	3,163.64	5,182.17
Intercept Imaginability	5.91	0.18	5.55	6.26	1.00	3,564.71	6,201.58
Correlation	0.78	0.01	0.77	0.80	1.00	21,228.02	14,548.74

Note. Estimates and errors indicate the mean and standard deviation of the posterior distribution, CI_{lower} and CI_{upper} display the lower and upper limits of the 95% credible interval.

Table S4

Output correlation model simultaneous presentation mode

Component	Estimate	Error	CI_{lower}	CI_{upper}	R-hat	Bulk ESS	Tail ESS
SD Intercept Likelihood	1.43	0.23	1.07	1.96	1.00	2,996.32	5,758.61
SD Intercept Imaginability	1.45	0.23	1.08	1.97	1.00	3,044.43	5,329.57
Intercept Likelihood	6.16	0.30	5.58	6.75	1.00	2,139.19	3,525.00
Intercept Imaginability	6.47	0.29	5.89	7.06	1.00	2,065.05	3,471.00
Correlation	0.84	0.01	0.83	0.85	1.00	12,120.72	12,860.65

Note. Estimates and errors indicate the mean and standard deviation of the posterior distribution, CI_{lower} and CI_{upper} display the lower and upper limits of the 95% credible intervals.

B: Difference between presentation modes

Figure S1

Difference between presentation modes

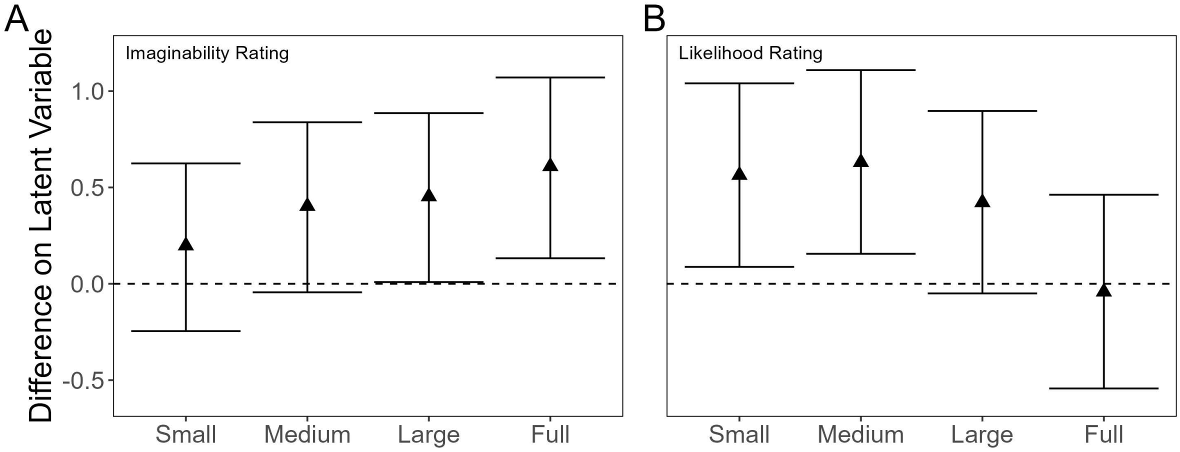

Note. Estimated difference in **A** the "imaginability" rating and **B** the "likelihood" rating between presentation modes. Error bars show the 95% credible interval. Positive values indicate higher ratings in the simultaneous presentation mode.

C: Control analysis temporal bias

C.1: Sequential presentation mode

Figure S2

Model estimates and differences between experimental halves for the sequential presentation mode

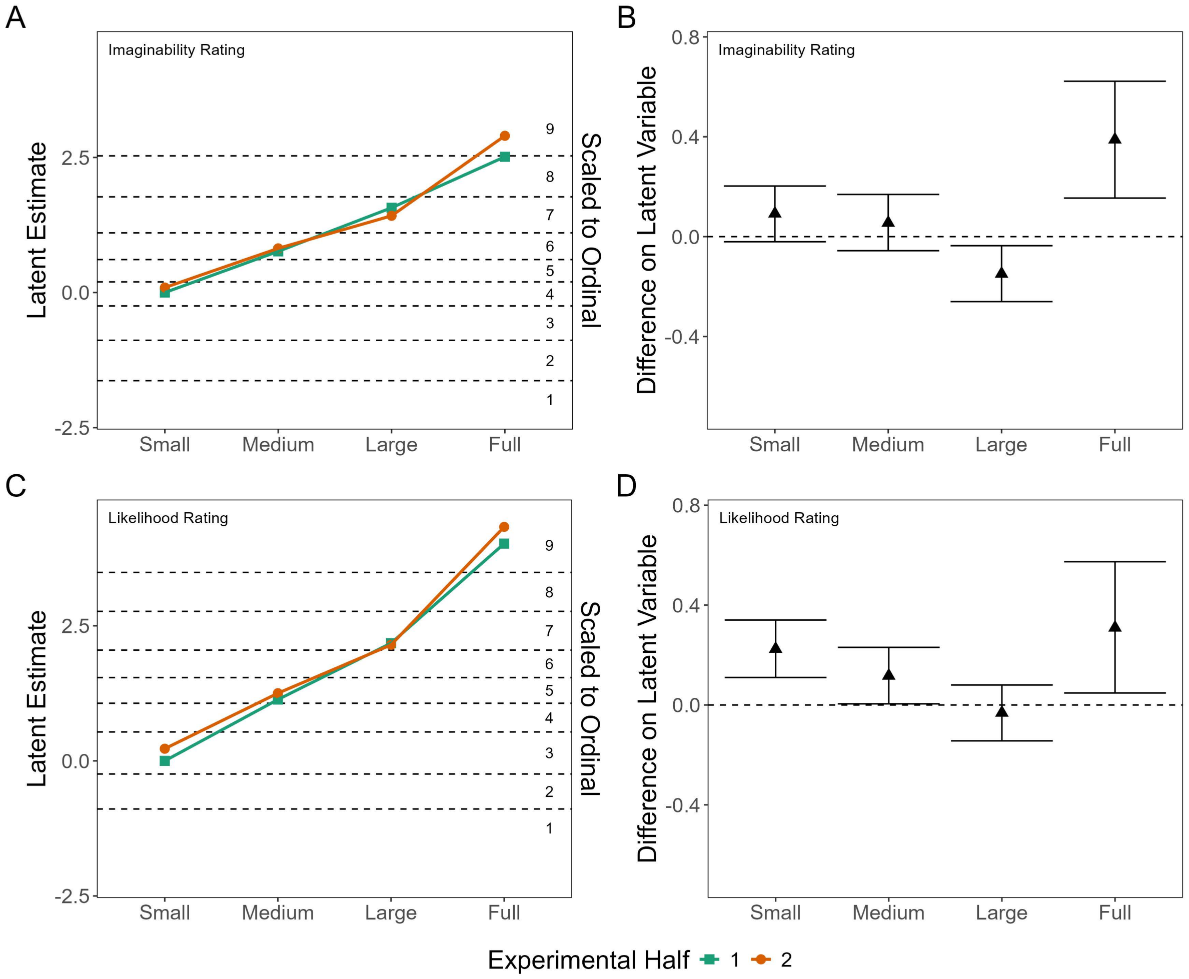

Note. **A** shows the model estimates and **B** the differences between the "imaginability" rating between experimental halves. Positive values in B indicate higher ratings in the second half of the experiment. **C** and **D** show the same as A and B, but for the "likelihood" rating. Error bars show the 95% credible interval.

Table S5

Comparison between experimental halves for the sequential presentation mode

Statement	Small	Medium	Large	Full
Imaginability	0.09 [-0.02, 0.20]	0.06 [-0.06, 0.17]	-0.15 [-0.26, -0.04]	0.39 [0.15, 0.62]
Likelihood	0.22 [0.11, 0.34]	0.12 [0.00, 0.23]	-0.03 [-0.14, 0.08]	0.31 [0.05, 0.57]

Note. Estimates of the difference between ratings in different experimental halves, depending on the occluder area for the sequential presentation mode. Values in brackets show the 95% credible interval. Positive values indicate higher ratings in the second half of the experiment.

C.2: Simultaneous presentation mode

Figure S3

Model estimates and differences between experimental halves for the simultaneous presentation mode

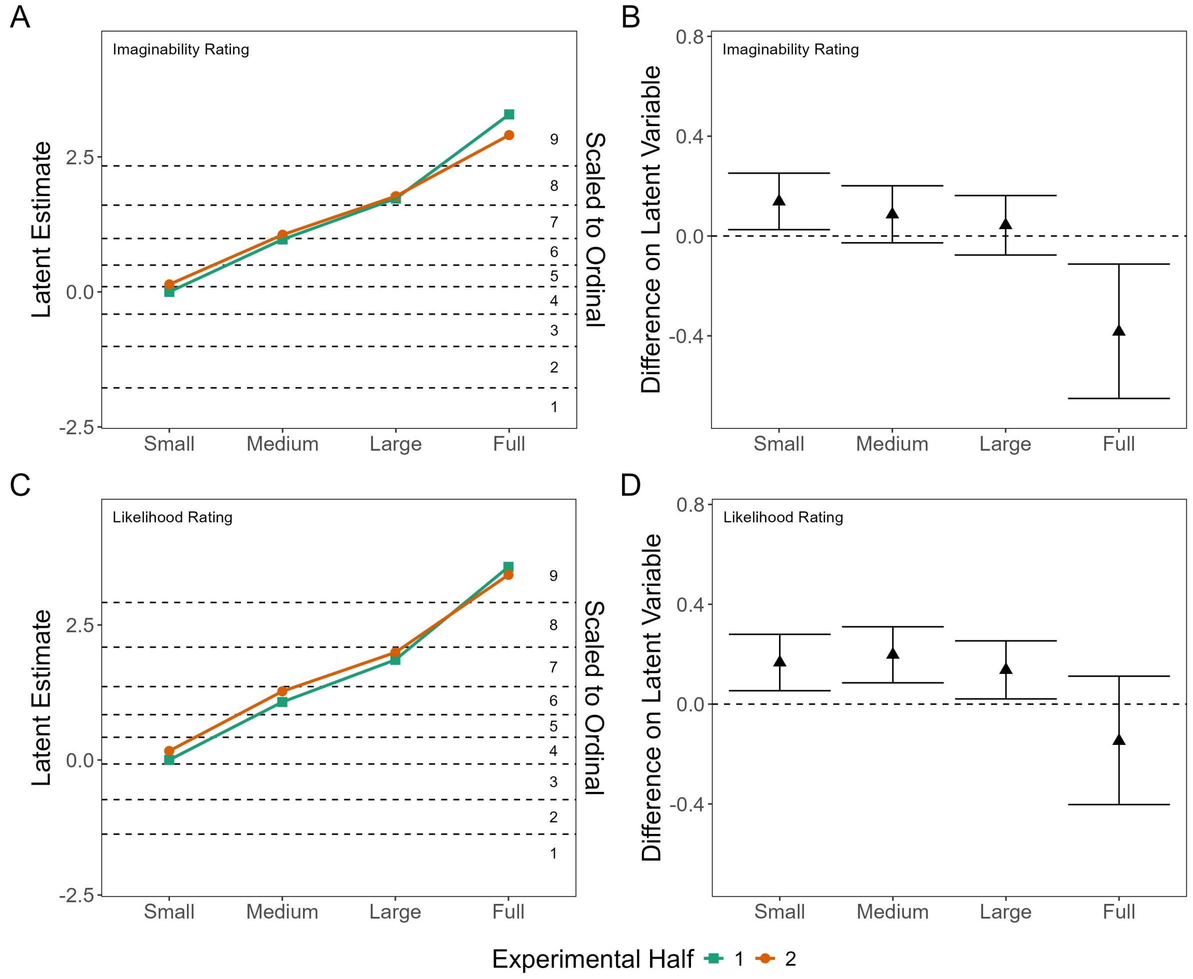

Note. **A** shows the model estimates and **B** the differences in the "imaginability" rating between experimental halves. Positive values in B indicate higher ratings in the second half of the experiment. **C** and **D** show the same as A and B, but for the "likelihood" rating. Error bars show the 95% credible interval.

Table S6

Comparison between experimental halves for the simultaneous presentation mode

Statement	Small	Medium	Large	Full
Imaginability	0.14 [0.03, 0.25]	0.09 [-0.03, 0.20]	0.04 [-0.08, 0.16]	-0.38 [-0.65, -0.11]
Likelihood	0.17 [0.05, 0.28]	0.20 [0.09, 0.31]	0.14 [0.02, 0.25]	-0.15 [-0.40, 0.11]

Note. Estimates of the difference between ratings in different experimental halves depending on the occluder area for the simultaneous presentation mode. Values in brackets show the 95% credible interval. Positive values indicate higher ratings in the second half of the experiment.
